# Inflammatory Kidney and Liver Tissue Response to Different Hydroxyethylstarch (HES) Preparations in a Rat Model of Early Sepsis

**DOI:** 10.1371/journal.pone.0151903

**Published:** 2016-03-17

**Authors:** Ralph C. Schimmer, Martin Urner, Stefanie Voigtsberger, Christa Booy, Birgit Roth Z’Graggen, Beatrice Beck-Schimmer, Martin Schläpfer

**Affiliations:** 1 Department of Surgery, University Hospital Zurich, Zurich, Switzerland; 2 Institute of Anesthesiology, University Hospital Zurich, Zurich, Switzerland; 3 Institute of Physiology and Zurich Center for Integrative Human Physiology, University of Zurich, Zurich, Switzerland; 4 Department of Anesthesiology, University of Illinois at Chicago, Chicago, Illinois, United States of America; University of Leicester, UNITED KINGDOM

## Abstract

**Background:**

Tissue hypoperfusion and inflammation in sepsis can lead to organ failure including kidney and liver. In sepsis, mortality of acute kidney injury increases by more than 50%. Which type of volume replacement should be used is still an ongoing debate. We investigated the effect of different volume strategies on inflammatory mediators in kidney and liver in an early sepsis model.

**Material and Methods:**

Adult male Wistar rats were subjected to sepsis by cecal ligation and puncture (CLP) and assigned to three fluid replenishment groups. Animals received 30mL/kg of Ringer’s lactate (RL) for 2h, thereafter RL (75mL/kg), hydroxyethyl starch (HES) balanced (25mL/kg), containing malate and acetate, or HES saline (25mL/kg) for another 2h. Kidney and liver tissue was assessed for inflammation. *In vitro* rat endothelial cells were exposed to RL, HES balanced or HES saline for 2h, followed by stimulation with tumor necrosis factor-α (TNF-α) for another 4h. Alternatively, cells were exposed to malate, acetate or a mixture of malate and acetate, reflecting the according concentration of these substances in HES balanced. Pro-inflammatory cytokines were determined in cell supernatants.

**Results:**

Cytokine mRNA in kidney and liver was increased in CLP animals treated with HES balanced compared to RL, but not after application of HES saline. MCP-1 was 3.5fold (95% CI: 1.3, 5.6) (p<0.01) and TNF-α 2.3fold (95% CI: 1.2, 3.3) (p<0.001) upregulated in the kidney. Corresponding results were seen in liver tissue. TNF-α-stimulated endothelial cells co-exposed to RL expressed 3529±1040pg/mL MCP-1 and 59±23pg/mL CINC-1 protein. These cytokines increased by 2358pg/mL (95% CI: 1511, 3204) (p<0.001) and 29pg/ml (95% CI: 14, 45) (p<0.01) respectively when exposed to HES balanced instead. However, no further upregulation was observed with HES saline. PBS supplemented with acetate increased MCP-1 by 1325pg/mL (95% CI: 741, 1909) (p<0.001) and CINC-1 by 24pg/mL (95% CI: 9, 38) (p<0.01) compared to RL. Malate as well as HES saline did not affect cytokine expression.

**Conclusion:**

We identified HES balanced and specifically its component acetate as pro-inflammatory factor. How important this additional inflammatory burden on kidney and liver function is contributing to the sepsis-associated inflammatory burden in early sepsis needs further evaluation.

## Introduction

Sepsis remains a major worldwide healthcare problem with consistently high mortality. Acute kidney injury (AKI) is a most prominent and severe complication of sepsis, occurring in 23% to 51% of patients depending on the severity of the sepsis [1] and causing more than 50% of the cases of AKI in patients treated in an ICU [2]. The pathophysiological understanding of sepsis-associated AKI has evolved from a simplistic hypovolemia to more complex concepts that better reflect the multifactorial nature of the condition. While sepsis-induced decrease in global renal blood flow (RBF) plays a major role in the development of AKI, there is now evidence that AKI may also occur under conditions of renal hyperperfusion [3]. However, hemodynamic changes associated with low cardiac output leading to renal hypoperfusion and ischemia-reperfusion injury do remain a major pathogenetic factor of sepsis-associated AKI. Consequently, fluid resuscitation continues to be a mainstay of treatment in septic AKI, and preservation of ‘physiological’ renal blood flow for prevention of further injury to the kidney seems imperative even in the absence of ischemia as initial pathogenic factor. For fluid resuscitation the choice of fluid, however, has been a widely debated topic, with type, timing and amount all being relevant factors potentially impacting on kidney function [4]. Synthetic and natural colloids as well as crystalloids are the most commonly used types of fluids, however adverse effects on the kidney attributed to hydroxyethyl starch (HES) preparations have been a concern for considerable time [5]. This lead to the rationale of this work supposing that HES could have a proinflammatory effect.

This study is specifically investigating potential differences in two different third-generation HES preparations (HES 130/0.42/6% saline and HES 130/0.42/6% balanced solution) compared to Ringer’s lactate (RL) for their pro-inflammatory potential in an early sepsis model in rats. With the advancing understanding of the pathogenesis of sepsis-induced organ dysfunction the importance of inflammatory changes has been emphasized [6] and it is consequently of considerable interest to identify a potential introduction of additional inflammatory changes induced by the administration of treatments like HES preparations. The study compares the effects of the two different HES preparations with RL on tissue expression of inflammatory markers in kidney and liver as well as the urinary markers creatinine and α-microglobuline in a cecal ligation and puncture (CLP) sepsis model in the rat. As the endothelium is the first compartment exposed to inflammatory stimuli in sepsis, the inflammatory response of endothelial cells exposed directly to the HES preparations was furthermore assessed *in vitro* using rat endothelial cells.

## Materials and Methods

### Animal experiment

The animal experiments and methods/ procedures applied have been approved and been in accordance with the local animal care committee (Veterinäramt des Kantons Zürich), approval number 132/2007.

Specific pathogen-free male Wistar rats (Charles River Laboratories, Germany) weighing 350g to 450g were used for the experiments. Animals were housed in standard cages at 22+/- 1°C under a 12/12h light/dark scheme. Food and water were available ad libitum.

For the induction of the sepsis rats were anesthetized using intraperitoneal ketamine (100mg/kg body weight) and xylazine (5mg/kg body weight). After shaving and disinfecting the lower quadrants of the abdomen a midline incision of approximately 4cm length was performed. The cecum was identified and the corresponding mesenteric membrane dissected. A cecal ligation was positioned consistently at the distal third, i.e. at 30–40% of the cecum. The perforation of the cecum was performed using a 18G needle, first puncturing the anti-mesenteric side, then continuing the needle penetration through the lumen to the second perforation on the mesenteric side. A small amount of fecal material was then gently extruded from both perforation holes. After repositioning the cecum within the abdominal cavity the abdomen was closed in two layers. Sham animals were treated in an identical fashion except from CLP.

Access for intravenous fluid resuscitation was obtained via a sterile 22G catheter (BD Insyte, Becton Dickinson SA, Madrid, Spain) inserted into the tail vein of the animals. Animals were monitored continuously and were kept at 37°C. Anesthesia/sedation was maintained by repeated subcutaneous administration of ketamine and xylazine (25mg/kg and 1.25mg/kg body weight) every 45min. Ketamine is a well known analgesic in order to minimize suffering of the animals.

Fluid resuscitation was performed using the following preparations and according to the following regimen: Ringer’s lactate (RL–Ringerlactat, B. Braun), HES 130/0.42/6% saline (HES saline—Venofundin 6%, B. Braun) or HES 130/0.42/6% balanced solution (HES balanced—Tetraspan 6%, B. Braun). The carrier solution of HES products is an electrolyte solution in case of HES saline it is normal saline, in case of HES balanced a balanced electrolyte solution containing apart form sodium, chloride, calcium, potassium, magnesium, acetate and malate. All animals received RL one hour after the procedure at a volume of 30mL/kg i.v. Two hours after the procedure, animals received either RL at a volume of 75mL/kg, HES saline or HES balanced at a volume of 25mL/kg.

The animals were sacrificed after 4h while under deep anesthesia with ketamine/xylazine. They were exsanguinated (by incising the abdominal aorta and inferior vena cava) and the heart was flushed with ice cold phosphate buffered saline (PBS), kidneys and liver were excised, snap-frozen in liquid nitrogen and stored at -80°C for RNA extraction.

**Fig 1:**
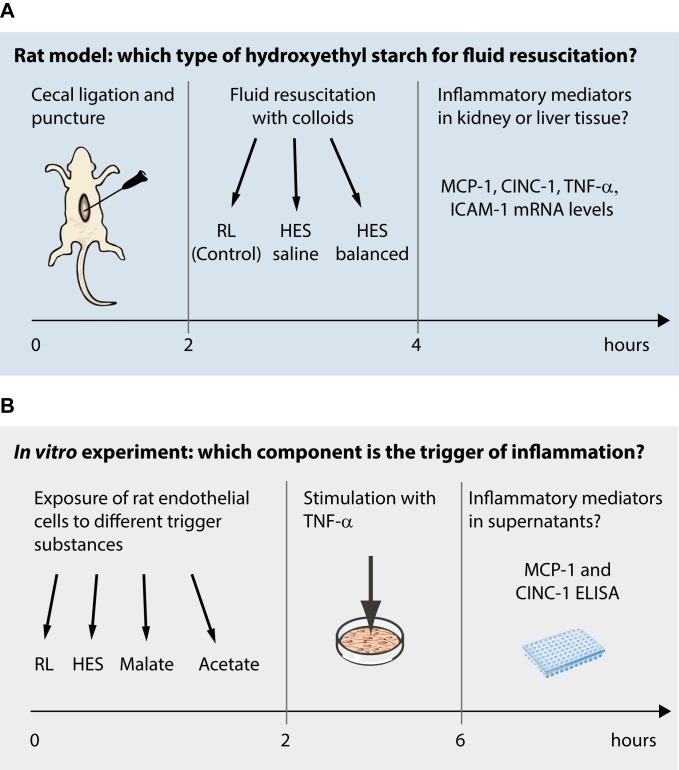
Experimental setting. Fig 1A gives an overview of the experimental animal setting and time line, Fig 1B displays the *in vitro* setup.

At least 4 animals were included in each sham group and 9 in each CLP arm of the study. The detailed experimental setup is displayed in **Fig 1A**.

### *In vitro* experiment

Rat pulmonary artery endothelial cells, a gift from Dr. Roscoe Warner (Assistant professor, Department of Pathology, University of Michigan at Ann Arbor, Ann Arbor, MI; Head: Dr. Peter A. Ward) [7, 8] were grown at 37°C in Dulbeco’s Eagle Medium (DMEM) completed with 10% fetal bovine serum (FBS), 1% penicillin/streptomycin and 1% 4-(2-hydroxyethyl)-1-piperazineethanesulfonic acid (HEPES, all from GIBCO, Carlsbad, CA). Confluent cells were incubated over night in starving medium containing 1% FBS. The next morning, cells were exposed to a 1:1 dilution of medium and either RL, HES saline or HES balanced, respectively. After a 2h the incubation solution was replaced by the same solution, completed with 0.5ng/mL tumor necrosis factor-α (TNF-α, BD Pharmingen, San Diego, CA). Control cells were exposed to phosphate-buffered saline only. After 6h cell supernatants were harvested and stored at -20°C for further analysis.

An analogue experiment was performed to determine the impact of the respective composition of the HES preparations (saline only or addition of malate and/or acetate). Ringer’s lactate was the reference volume replacement. Concentrations used were 24mmol sodium acetate, 5mmol sodium malate or a mixture of 24mmol sodium acetate plus 5mmol sodium malate. The amount of acetate and malate was chosen according to the concentrations of acetate and malate in HES balanced. An overview of the experimental setting is given in **Fig 1B**.

### Assessment of inflammation using enzyme-linked immunosorbent assay (ELISA) and real time polymerase chain reaction (PCR)

To be able to compare our results with other animal studies focusing on systemic or inflammatory processes we decided to focus on inflammatory mediators. Furthermore, determination of these markers allowed us to differentiate between systemic effects and inflammatory scenarios in the various tissues, where the cytokines are expressed as well. This aspect of compartmentalization seems to be essential.

Expression of MCP-1, CINC-1, TNF-α and intercellular adhesion molecule-1 (ICAM-1) mRNA was determined in kidney and liver tissue by real-time PCR. Tissue was homogenized using MagNA Lyser Green Beads (Roche Diagnostics, Basel, CH) and a Precellys Homogenizer for 1x30sec at 6800rpm. Total RNA was purified using the RNeasy mini Kit (Qiagen AG, Hombrechtikon, CH). 0.5μg RNA was used for reverse transcription using the Taqman Reverse Transcription Kit (ABI, Life Technogolies, CH). Quantitative PCR was performed using the PCR Probe Mastermix and labeled probes (both from Roche) and primers (Microsynth, Balgach, CH), designed for MCP-1, CINC-1, TNF-α, ICAM-1 and the housekeeping gene 18S.

The final reaction volume was 15μl using a Gene Amp 5700 System (ABI, Life Technologies, CH). The comparative Ct method was used for quantification of the gene expression. Ct values of the samples were normalized to 18S.

The cytokines monocyte chemoattractant protein-1 (MCP-1) and cytokine-induced neutrophil chemoattractant-1 (CINC-1) in cell supernatants were determined by sandwich ELISA technique according the manufacturer’s protocol (both from R&D Systems, Abingdon, UK).

### Creatinine and α-microglobulin determination

Creatinine was assessed using a DriChem 4000i Analyzer (Fuji Film, Tokio, Japan) and the corresponding DriChem slides (Fuji) according the manufacturer’s instruction.

Alpha-microglobulin determination took place by an ELISA-kit according the manufacturer’s protocol (Hölzel Diagnostika, Cologne, Germany).

### Statistical analysis

Statistical analysis was performed using R, Version 3.2.2 (R Development Core Team, 2015), with the packages lme4 [9], lmerTest [10], and ggplot2 [11]. Linear mixed model analyses were used to assess the influence on tissue inflammatory mediator expression by the different types of treatment. Exposure to RL has been defined as the reference category. The tabular results show coefficients and corresponding 95% confidence intervals of the mixed models. Figures illustrate boxplots with medians and quartiles. Whiskers represent 5% and 95% confidence intervals. A p-value <0.05 was considered significant.

## Results

With the aim to study a potential treatment-associated inflammatory effect in very early stages of AKI the present short-term model of CLP was chosen. Correspondingly, mild but consistent increases in inflammatory markers were observed in the CLP RL-treated compared to sham-operated RL-treated animals. In the kidney, MCP-1 and CINC-1 expression increased by a 2.8- and 3.5-fold compared to sham-operated animals (both p<0.01, **Table 1.** This was less pronounced for TNF-α levels in CLP animals (1.4-fold increase, p<0.01). Intercellular adhesion molecule-1 was not significantly more expressed in CLP than in sham animals.

**Table 1:** Influence of CLP and different fluid resuscitation procedures on inflammatory mediator expression (mRNA) in kidneys.

Dependent variables	Independent variables			R^2^
	CLP	HES balanced	HES saline	
MCP-1, fold change	2.8 (0.7, 4.8) ^2^	3.5 (1.3, 5.6) ^2^	-0.2 (-2.4, 1.9)	0.329
CINC-1, fold change	3.5 (1.1, 5.8) ^2^	1.8 (-0.6, 4.3)	0.6 (-1.69, 2.9)	0.201
TNF-α, fold change	1.4 (0.4, 2.4) ^2^	2.3 (1.2, 3.3) ^1^	-0.3 (-1.2, 0.7)	0.415
ICAM-1, fold change	1.2 (0.2, 2.2) ^3^	2.2 (1.2, 3.2) ^1^	-0.7 (-1.0, 0.9)	0.394

Exposure to Ringer’s lactate solution has been defined as the reference group in the mixed model. The table shows coefficients with corresponding 95% confidence intervals. CINC-1: cytokine-induced neutrophil chemoattractant 1; CLP: cecal ligation and puncture; HES: hydroxyethyl starch; ICAM-1: intercellular adhesion molecule-1; MCP-1: monocyte chemotactic protein 1; TNF-α: tumor necrosis factor-α.

^1^ p≤ .001

^2^ p≤ .01

^3^ p≤ .05.

The inflammatory response of CLP compared to sham animals was similarly observed in liver tissue: a 51.7-fold increase of MCP-1 was seen in the liver of CLP animals (**Table 2**). The CLP procedure had a marginal effect on CINC-1 mRNA expression (upregulation by a 0.6-fold, p<0.05) and no quantifiable effect on TNF-α and ICAM-1 levels.

**Table 2:** Influence of CLP and different fluid resuscitation procedures on inflammatory mediator expression (mRNA) in the liver.

Dependent variables	Independent variables			R^2^
	CLP	HES balanced	HES saline	
MCP-1, fold change	51.7 (3.5, 99.9) ^3^	118.7 (63.8, 173.5) ^1^	-5.0 (-53.5, 43.5)	0.268
CINC-1, fold change	0.63 (0.1, 1.2) ^3^	0.6 (0.0, 1.3)	-0.5 (-1.1, 0.1)	0.428
TNF-α, fold change	49.9 (-26.7, 126.6)	134.4 (47.2, 221.6) ^2^	-6.4 (-83.4, 70.8)	0.344
ICAM-1, fold change	196.8 (-52.1, 445.7)	512.3 (229.7, 795.0) ^1^	-24.5 (-278.0, 229.0)	0.295

Exposure to Ringer’s lactate solution has been defined as the reference group in the mixed model. The table shows coefficients with corresponding 95% confidence intervals. CINC-1: cytokine-induced neutrophil chemoattractant 1; CLP: cecal ligation and puncture; HES: hydroxyethyl starch; ICAM-1: intercellular adhesion molecule-1; MCP-1: monocyte chemotactic protein 1; TNF-α: tumor necrosis factor-α.

^1^ p ≤ .001

^2^ p ≤ .01

^3^ p ≤ .05.

This early phase-CLP model resulted in a mild consistent inflammatory tissue response in kidney and liver corresponding to early systemic changes as a consequence of the local intra-abdominal inflammatory process. It is noteworthy that despite this relatively mild inflammation the sensitivity of the model was sufficiently high to distinguish different degrees of inflammatory reactions to the various volume repletion treatments.

### Different inflammatory response to various fluid resuscitation treatments in the kidney

**Fig 2:**
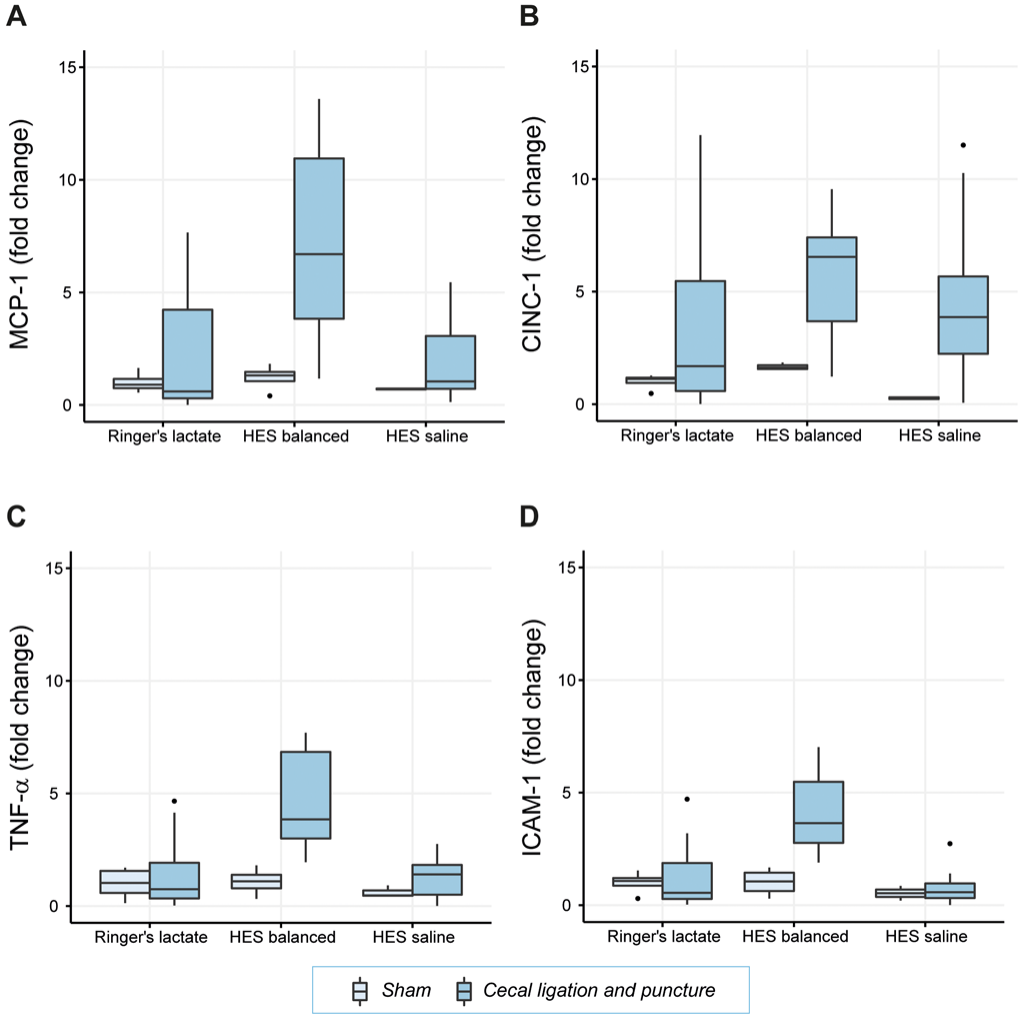
Inflammatory mediator expression (mRNA) in kidney tissue. Relative mRNA levels of the inflammatory cytokines MCP-1 (Fig 2A), CINC-1 (Fig 2B), TNF-α (Fig 2C), and ICAM-1 (Fig 2D) are shown. Results are presented as boxplot figures with medians and quartiles. Whiskers represent 5% and 95% confidence intervals.

In CLP animals expression of inflammatory mediators upon administration of HES saline was similar to animals treated with RL. In contrast, administration of HES balanced led to an additional 3.5-fold increase of MCP-1 (p< 0.01), a 2.3-fold increase of TNF-α (p<0.001) and a 2.2-fold increase of ICAM-1 (p ≤ 0.001) (**Table 1**, **Fig 2**).

**Fig 3:**
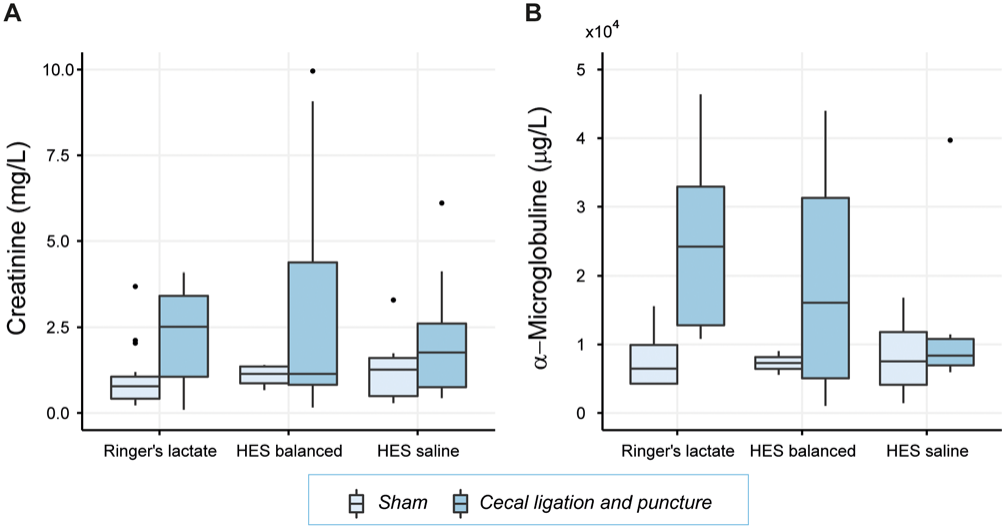
Creatinine and α-microglobuline concentration in urine. Kidney function was assessed by creatinine (Fig 3A) and α-microglobuline (Fig 3B). Results are presented as boxplot figures with medians and quartiles. Whiskers represent 5% and 95% confidence intervals.

In order to further evaluate a possible impact of the administration of different HES preparations on kidney function creatinine and α-microglobuline levels in the urine were measured (**Table 3**, **Fig 3**). In rats undergoing CLP creatinine increased by 1.23mg/dL, (p<0.01) and α-microglobuline by 11μg/mL (p<0.05) compared to sham-operated animals. A significant influence of the different fluid resuscitation regimens was not apparent.

**Table 3:** Influence of different fluid resuscitation procedures on excretion of creatinine and α-microglobuline in urine.

Dependent variables	Independent variables			R^2^
	CLP	HES balanced	HES saline	
Creatinine, mg/dL	1.23 (0.47, 1.98) ^1^	0.56 (-0.47, 1.59)	-0.03 (-0.84, 0.78)	0.143
α-microglobuline, μg/mL	11 (3, 20) ^1^	-3 (-14, 8)	-5 (-15, 4)	0.234

Exposure to Ringer’s lactate solution has been defined as the reference group in the mixed model. CLP: cecal ligation and puncture; HES: hydroxyethyl starch. The table shows coefficients with corresponding 95% confidence intervals.

^1^ p ≤ .01.

### The inflammatory response to different fluid resuscitation treatments in the liver

**Fig 4:**
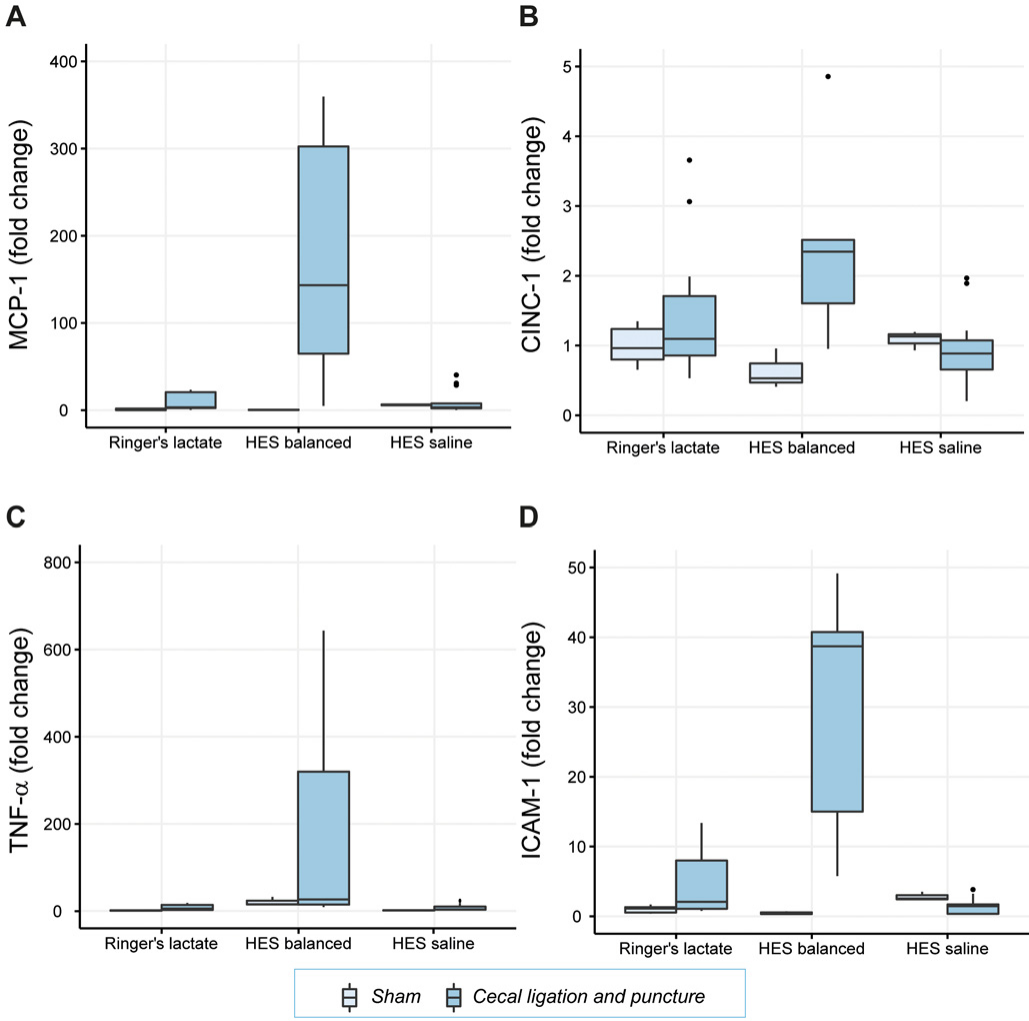
Inflammatory mediator expression (mRNA) in liver tissue. Relative mRNA levels of the inflammatory cytokines MCP-1 (Fig 4A), CINC-1 (Fig 4B), TNF-α (Fig 4C) and ICAM-1 (Fig 4D) are shown. Results are presented as boxplot figures with medians and quartiles. Whiskers represent 5% and 95% confidence intervals.

Cecal ligation and puncture animals showed a significant increase in hepatic mRNA expression of MCP-1 by 118.7-fold (p<0.001), of TNF-α by 134.4-fold (p<0.01), and of ICAM-1 by a 512.3-fold (p<0.001) when treated with HES balanced compared to CLP animals with application of RL (**Table 3**, **Fig 4**). This difference was not seen when animals were given HES saline. Overall and similar to the results in kidney tissue, the exposure to HES balanced resulted in an accentuation of the inflammatory response in septic animals.

### Determination of the inflammation-triggering component of volume repletion treatment with HES balanced *in vitro*

**Fig 5:**
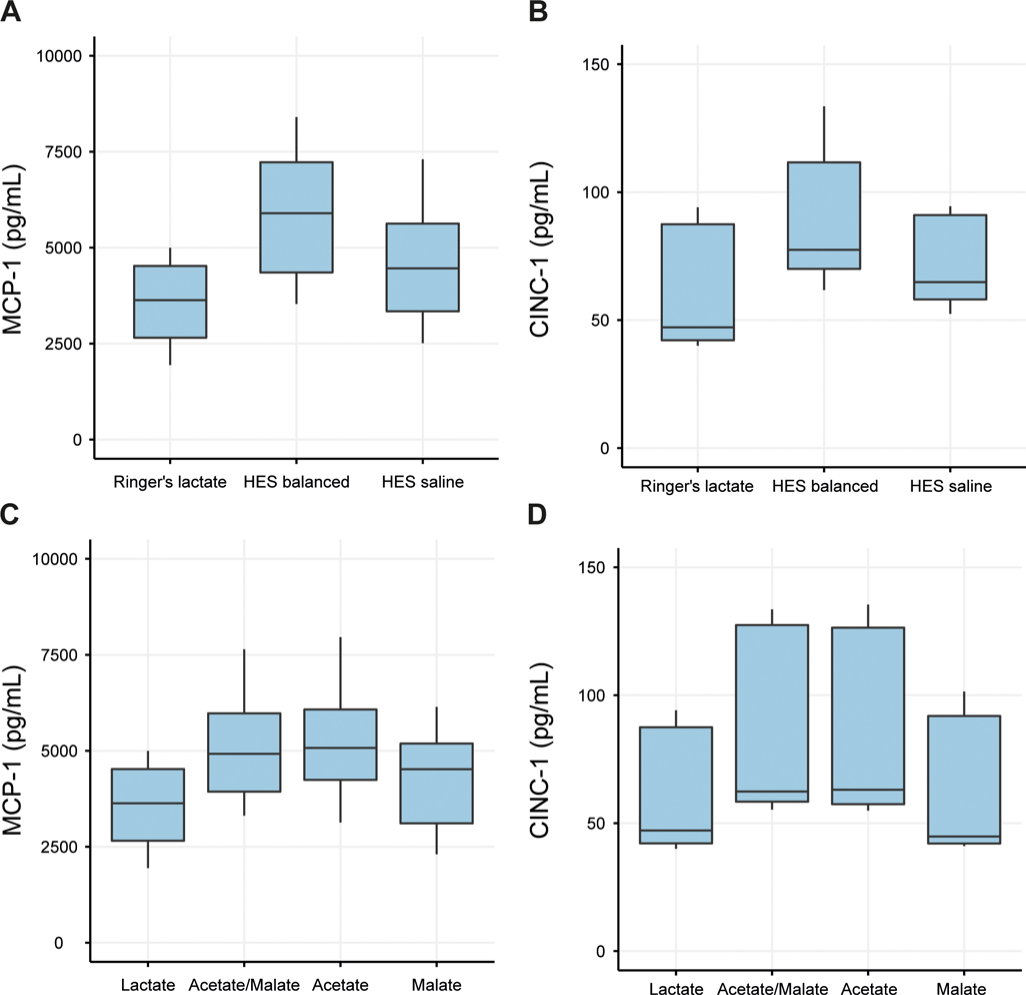
Inflammatory response of rat pulmonary endothelial cells to fluid formulations or components and subsequent stimulation. Levels of the inflammatory cytokines MCP-1 (Fig 5A and 5B) and CINC-1 (Fig 5C and 5D) after exposure to fluid formulations (RL, HES balanced and HES saline, Fig 5A and 5C) and anions present in balanced solutions (5B and 5D). Results are presented as boxplot figures with medians and quartiles. Whiskers represent 5% and 95% confidence intervals.

The *in vivo* results demonstrating a significantly increased inflammatory response in both kidney and liver tissue of animals with HES balanced volume resuscitation in an early septic condition raised the question about a potentially causative component of the HES balanced volume repletion treatment. For further exploration, the *in vivo* model was thus translated into an *in vitro* model of endothelial inflammation for a detailed examination. In analogy to the animal experiments, an inflammatory response in endothelial cells was induced by stimulation with TNF-α, and expression levels of MCP-1 and CINC-1 were assessed. Cells exposed to TNF-α and RL expressed 3529±1040pg/mL MCP-1 and 59±23pg/mL CINC-1 protein. As illustrated in **Table 4**exposure to HES balanced increased the secretion of inflammatory mediators in TNF-α-stimulated endothelial cells by an additional 2358pg/mL for MCP-1 (p<0.001) (**Fig 5A**) and an additional 29pg/mL for CINC-1 (p<0.01) (**Fig 5B**). The levels of MCP-1 and CINC-1 were not significantly affected by HES saline in TNF-α-stimulated endothelial cells (**Table 4**).

**Table 4:** Influence of different fluid solutions inflammatory mediator secretion (protein) of rat endothelial cells.

Ingredients	HES balanced	HES saline	R^2^
MCP-1, pg/mL	2358 (1511, 3204) ^1^	1003 (156, 1850)	0.329
CINC-1, pg/mL	29 (14, 45) ^2^	12 (-3, 28)	0.235

Exposure to Ringer’s lactate solution has been defined as the reference group in the mixed models. The table shows coefficients with corresponding 95% confidence intervals. CINC-1: cytokine-induced neutrophil chemoattractant-1; HES: hydroxyethyl starch; MCP-1: monocyte chemotactic protein-1.

^1^ p ≤ .001

^2^ p ≤ .01.

In order to further characterize a potential association with the electrolyte composition of the volume resuscitation treatments the cells were either exposed to RL or to PBS with or without addition of acetate and/or malate. Exposure to HES balanced or acetate, resulted in higher MCP-1 concentrations in the supernatants of the endothelial cells. Compared to cells exposed to TNF-α and RL, mean MCP-1 levels were elevated by 842pg/mL after incubation with HES balanced (p<0.01, Fig 5A) and by 1059pg/mL after exposure to acetate-containing PBS (p<0.01, Fig 5C). No significant effect on MCP-1 secretion was observed after incubation with HES saline or malate in endothelial cells (**Table 5**). Consistent with the results for MCP-1, HES balanced further increased CINC-1 expression by 29pg/mL (p<0.01), acetate by 21pg/mL (p<0.01) compared to RL (Fig 5D).

**Table 5:** Influence of specific solution components on inflammatory mediator secretion of rat endothelial cells.

Ingredients	Acetate	Malate	R^2^
MCP-1, pg/mL	1325 (741, 1909) ^1^	291 (-293, 875)	0.199
CINC-1, pg/mL	24 (9, 38) ^2^	0 (-14, 15)	0.143

Exposure to Ringer’s lactate solution has been defined as the reference group in the mixed model. The table shows coefficients with corresponding 95% confidence intervals. CINC-1: cytokine-induced neutrophil chemoattractant 1; MCP-1: monocyte chemotactic protein 1.

^1^ p ≤ .001

^2^ p ≤ .01.

## Discussion

In summary our *in vivo* results show increased levels of inflammation for the cecal ligation and puncture model. This was uninfluenced by RL and HES saline, but was further accentuated by HES balanced.

Similar observations could be made in endothelial cells *in vitro*: TNF-α induced an inflammation in rat endothelial cells, which was uninfluenced by HES saline, but was even more accentuated in the presence of HES balanced. Testing the solution’s electrolyte composition, acetate in the concentration found in HES balanced lead to a more pronounced inflammation while malate did not.

Sepsis remains a major worldwide healthcare problem with consistently high mortality. The condition can be generally described as the occurrence of an infection together with a systemic response to and manifestation of the infection [12]. The body’s systemic response to a pathogen may result in conditions of various levels of severity that have been categorized in three stages [12, 13]: sepsis, severe sepsis and septic shock. Sepsis is characterized by a Systemic Inflammatory Response Syndrome (SIRS), the presence of at least 2 out of its 4 defining criteria and a proven or suspected infection. Severe sepsis further comprises sepsis-induced organ dysfunction or tissue hypoperfusion characterized by e.g. elevated lactate, oliguria or hypotension. Septic shock represents the most severe stage with persistent hypotension and multi-organ dysfunction and failure. Pathophysiologically, sepsis is understood as a syndrome characterized by complex series of events with immune system activation resulting in pro- and anti-inflammatory reactions, humoral and cellular responses, abnormalities in microcirculation predisposing to impaired general oxygen delivery, tissue hypoxia, multiorgan dysfunction and ultimately death [14, 15].

Sepsis-induced organ dysfunction and failure are most severe progression steps of the initial systemic inflammatory response and usually the cause of death when severe sepsis and shock are present. Target organs include the lungs, kidneys and liver. Sepsis-associated AKI reaches a mortality of 70% compared to a 45% mortality rate in AKI alone [1] and thus constitutes a severe medical problem.

The specific mechanisms leading to organ failure in sepsis and also the varying degree of vulnerability of different organs are still not well understood. The systemic inflammatory response, tissue hypoperfusion associated with hypotension[16, 17] and disseminated intravascular coagulation (DIC) [18] seem to represent the major causative elements leading to organ dysfunction [18].

The benefit-risk profile of fluid resuscitation as therapeutic intervention might be associated with an inflammatory response in itself and may thus represent a potentially aggravating factor in the development of the complex pathophysiologic situation in sepsis.

The reported safety profile of HES preparations has been characterized by adverse effects predominantly on the coagulation system and renal function [19]. HES preparations have seen development with decreasing molecular weights and molar substitution to arrive at the current third generation preparations in order to improve their benefit-risk profile with reduced adverse effects on these two organ systems. The initially higher molecular weight was aiming at particularly long residence time in the circulation, an effect that receives less emphasis today in favor of better pharmacodynamic control and potentially reduced adverse effects.

The inflammatory response to tissue injury has been a main research focus of the authors, in particular in the lung [20–23]. In the present study, the research question centered on inflammatory changes in an early *in vivo* sepsis model induced by cecal ligation and puncture and investigating kidney and liver tissue following various forms of fluid resuscitation as a hallmark of sepsis treatment in order to improve hypotension and associated organ hypoperfusion. As a secondary question the hypothesized inflammatory response differences to the various fluid resuscitation regimens were to be analyzed for a potential association with distinct fluid components. An *in vitro* model of endothelial inflammation was chosen to discern differences in the inflammatory response to the administration of different components.

We found a significantly more pronounced inflammatory mediator expression in animals treated with HES balanced compared to those receiving HES saline or RL lactate. This effect could be successfully transferred, reproduced and further analyzed *in vitro* in a model of inflamed rat endothelial cells. Our analysis revealed that acetate but not malate or HES saline are associated with an increased expression of inflammatory mediators.

There is a still on-going debate about the use of colloids in critically ill patients: concerns have been raised with regard to possible adverse outcomes in patients receiving HES solutions, especially in septic patients [24–26] and even a withdrawal of the marketing authorization for HES has been demanded [27]. However, HES continues to be widely used for intravascular volume maintenance or augmentation in daily clinical routine [28]. In contrast to septic patients, no differences in the incidence of death or acute kidney failure has been found in surgical and trauma patients receiving 6% HES [29], for which reason a more nuanced view on the use of HES solutions has been proposed [30].

In this model of CLP-induced septic peritonitis in rats, the research focus was on the very early expression of prognostic inflammatory mediators impacting on severity and outcome of sepsis [31, 32]. No effect of HES was observed on inflammatory mediator expression in kidney and liver tissue. Interestingly a clear difference between HES balanced and HES saline has been found. HES balanced provoked a significantly more pronounced expression of inflammatory mediators compared with HES saline. This is in line with results from an earlier study investigating the effects of balanced and non-balanced colloids in acute endotoxemia [23]. In the same study, it has been hypothesized that balanced solutions containing acetate and malate could be specifically associated with conditions of severe inflammation. The present data now provides further experimental evidence that in particular acetate appears to be associated with an aggravation of the inflammatory response. This observation is corroborated by data showing other pro-inflammatory and also myocardial depressant and hypoxemia promoting characteristics [33–35] induced by acetate which led for example to the discontinuation of its use in fluids for renal replacement therapy.

Functional renal testing does not support our findings on the level of inflammatory mediators. This is likely due to the fact that inflammation-induced functional impairment in organs is observed at a much later time point. We tried to differentiate between an inflammatory process, which is induced by application of the balanced solution, and a possible inflammatory-toxic effect, which might be seen immediately in changes in creatinine and alpha-microglobulin values.

**Strengths and limitations of the experimental approach.** Our experimental approach has several strengths starting with the use of an early stage *in vivo* model of polymicrobial sepsis. In addition to confirming previous results in a different sepsis model [23], we do not only investigate differences in the inflammatory response to several fluid resuscitation solutions in vivo but furthermore also identify individual components associated with the stronger inflammatory response to HES balanced in our *in vitro* approach. A certain limitation of the study might is that this–like all models—represents a simplification of a scenario in the patient. This however allows discriminating factors impacting on a certain diseases in smaller study groups.

**Conclusions and outlook.** The data presented in this experimental study indicate that not HES balanced per se but specifically acetate in HES balanced is a component contributing to a more pronounced inflammatory response to the administration of HES balanced in an early sepsis model. This additional pro-inflammatory element could potentially add to the overall burden of the local and systemic inflammatory response in sepsis and therefore may impact negatively on the course of the disease.

## Supporting Information

S1 File Dataset. (XLSX)
